# Birth asphyxia and its associated factors among newborns in public hospital, northeast Amhara, Ethiopia

**DOI:** 10.1371/journal.pone.0226891

**Published:** 2019-12-20

**Authors:** Abay Woday, Ayesheshim Muluneh, Christine St Denis

**Affiliations:** 1 Samara University, College of Medical and Health Sciences, Department of Public Health, Afar Region, Samara, Ethiopia; 2 College of Health Science, Amhara Region, Dessie, Ethiopia; 3 Sickkids Hospital, Toronto, Canada; Addis Ababa University School of Public Health, ETHIOPIA

## Abstract

**Background:**

Birth asphyxia is a leading cause of infant morbidity and mortality in developing nations, such as Ethiopia. Though Ethiopia has made considerable achievement in the reduction of under-five mortality rate, the neonatal mortality burden has not experienced the same reduction, which may be attributed to birth asphyxia. Thus, this study attempts to assess the prevalence and associated factors of birth asphyxia among newborns in public hospitals in the northeastern Amhara region, Ethiopia.

**Methods:**

An institution-based cross-sectional study was conducted on 357 births from 1^st^ April to 2^nd^ May 2018. The sample size was proportionally allocated to randomly selected three public hospitals namely, Dessie referral hospital, Debre Berhan referral hospital, and Woldia general hospital. The allocation was made by taking the average number of deliveries given in each hospital six months before the data collection period. Using the delivery registration of hospitals a systematic random sampling technique was used to get all study participants. The diagnosis of birth asphyxia was confirmed based on the physician’s diagnosis of an APGAR score < 7 in the 1^st^ and 5^th^ minutes of birth. A pretested and structured questionnaire was used to collect data. Variables with p-values < 0.25 in the bivariable analysis were entered into a multivariable logistic regression analysis. A statistical significant level was declared at a p-value of <0.05.

**Results:**

The prevalence of birth asphyxia was found to occur 22.6% of the time [95% CI 19.2% - 26.4%] in the first minute of birth. In the multivariable logistic regression being primipara [AOR = 3.77: 95% CI 1.86, 7.65], presented with complicated labor [AOR = 3.45: 95% CI 1.58, 7.49], premature rupture of membrane [AOR = 3.85: 95% CI 1.76, 8.44) and having blood-stained amniotic fluid at birth [AOR = 5.02: 95% CI 1.69, 14.87] were the independent predictors of birth asphyxia.

**Conclusion:**

The study revealed that birth asphyxia is a common newborn complication in the Amhara region. Integrated mitigation measure to reduce neonatal mortality in the Amahar region should give due attention to primipara women and for these high-risk pregnancies in order for the region to achieve national and global commitment to have sustainable change in women and neonatal health.

## Introduction

The World Health Organization (WHO) defines birth asphyxia as inadequate oxygen perfusion to vital organs, generally, caused by a failure to initiate and sustain breathing at birth [1]. Additionally, the American College of Obstetricians and Gynecologists and the American Academy of Pediatrics expand on the definition of neonatal asphyxia if the following conditions are fulfilled: umbilical cord arterial pH <7; APGAR score of 0–3 for longer than 5 min; neurological manifestations such as seizures, hypercapnia, metabolic acidosis and hypoxic-ischemic encephalopathy [2, 3].

Globally 2.5 million children died in the first month of life which contributed 47% of all child deaths under the age of 5-years and 54 percent of all under-five deaths occur during the neonatal period among African babies [4]. However, more than two-thirds of newborns could be saved through existing maternal and child health programs though most deaths happened at home and they are invisible to the national and regional policies and programs [5]. Worldwide, about 25% of all neonatal deaths are caused by birth asphyxia [6]. In Ethiopia, in 2015, it was the first cause of neonatal deaths (31.6%), followed by prematurity (21.8%) and sepsis (18.5%) [7]. More specifically, the Amhara region of Ethiopia contributes the highest neonatal deaths rates (47/1000 live births) as compared to the other nine regions found in Ethiopia [8].

Furthermore, the prevalence of birth asphyxia varies across the globe and Africa contributes nearly 50% of the total [9–12]; its prevalence ranges between 3.1% -56.9% in Ethiopia [13–15]. Previously conducted studies identified various contributing factors of birth asphyxia such as antepartum risk factors (i.e. maternal age, maternal education, pre-eclampsia and primi-gravida,) intrapartum risk factors (i.e. breech presentation, mode of delivery and maternal fever, and fetal risk factors (i.e. pre-term babies, fetal distress and baby weight) [16–19].

Presently, the Sustainable Development Goals of 2030, which combine multisystem strategies at global and national levels, have three main focuses to ensure healthy lives and promote wellbeing for all at all ages. Of these goals, one main objective is to reduce the neonatal mortality rate to lower than 12 per 1,000 live births [20–22].

Despite the implementation of various strategies and interventions at combating under-five children, infant and neonatal mortality rates [4, 6, 23, 24], birth asphyxia remains one of the most common causes of neonatal mortality and continues to be a major public health concern, especially in developing nations such as Ethiopia. Moreover, limited studies have been carried out in Ethiopia to date to generate information for action regarding birth asphyxia. Therefore, this study was intended to determine the prevalence and associated factors of birth asphyxia among newborns in the northeast Amhara region of Ethiopia.

## Methods

### Study settings and participants

An institution-based cross-sectional study was conducted from 1^st^ April to 2^nd^ May2018 in order to assess the prevalence and associated factors of birth asphyxia among newborns in public hospitals found in the northeastern part of Amhara regional state namely: Dessie referral hospital, Debre Birhan referral hospital and Woldia District Hospital. The hospitals are located 130kms, 401kms, and 421kms away from the capital city, Addis Ababa, respectively. Combined, these hospitals serve more than two million people living in the catchment areas.

In this study, all neonates with mothers, born with a gestational age ≥ 28 weeks, and delivered live births in selected hospitals during the pre-determined period of study were included in the research. However, mothers who were seriously ill during the study period, neonates who were suffering from major congenital anomalies or syndromes, and those neonates immediately transferred to advanced care before the five-minute APGAR score evaluation were excluded from this study.

The sample size was calculated using a single population proportion formula. The prevalence of perinatal asphyxia was taken as 33% from the previous study conducted in Jimma, Southwest Ethiopia [25] with the basic assumptions of 95% confidence level (the Critical value Zα/2 = 1.96), 5% margin of error and the researchers added 5% to compensate the non-responses. Then, the calculated sample size becomes (340**×**0.05 + 340) = 357.

$$n=\frac{{\left(Za/2\right)}^{2}(P)(1-P)}{d^{2}}=340$$

Where: n = the required sample size, Z α/2 = the standardized normal distribution curve value for the 95% confidence level, P = the proportion of birth asphyxia among the general population, and d = degree of precision (the margin of error between the sample and population).

There are eight hospitals in the northeastern Amhara region. Of those, three hospitals were randomly selected for this study, namely, Dessie referral hospital, Debre Berhan referral hospital, and Woldia general Hospital. The number of study participants for each hospital was proportionally allocated based on the monthly average number of delivery in each hospital. A systematic sampling technique with every 3^rd^ interval was used to enroll study participants in each delivery unit of the hospitals.

### Data collection tools and procedures

The data collection tool, which this survey used, was adapted and modified from various studies conducted in developing countries [11, 15, 19] and contextualized to Ethiopia. The questionnaire was developed based on sociodemographic characteristics, obstetric factors (fetal and maternal factors in prenatal, intrapartum and postpartum periods) and health service-related questions. The data collection instrument was translated into the local language (Amharic) and back to English to keep consistent. To ensure the reliability of APGAR scoring, two days of training was provided for data collectors and supervisors. In addition, the questionnaire was pretested on 5% of the samples at non-selected public hospitals, where a panel of experts verified the content validity of the instruments, and based on the feedback the required revisions were made.

Data was collected by trained midwives using a structured interviewer-administered questionnaire and chart audit. Data collection commenced during the second stage of labor and ended by the fifth minute of the postpartum period for newborn related data. However, the mothers were interviewed within 4 hours of giving birth since mothers who had given normal births were discharged home after six hours of stay in the three hospitals.

Birth asphyxia is the inability of newborn to initiate and sustain adequate respirations after delivery within 1^st^ and 5^th^ minutes of birth and ending with an APGAR score < 7.[12]. Birth weight of the newborn was measured using calibrated weight measuring scale placed in labor ward, in which the measurement result was rounded into the nearest value of 100grams. Maternal nutritional status was measured using Mid-Upper-Arm-Circumference (MUAC). There are certain controversies upon the cut-off value of MUAC. Some scholars agreed if MUAC is less than 21cm, it is severe acute malnutrition among pregnant women while others agreed that if MUAC<22, it is Short Acute Malnutrition (SAM) among pregnant women, and others if MUAC is less than 23cm, it is more conservative [26–28]. Thus, in this study, MUAC<22cm was taken as the cut-off value of malnutrition among pregnant women.

### Study variables

The dependent variable was birth asphyxia. The independent variables included in this study were: (1) maternal and newborn sociodemographic characteristics (age, marital status, sex of the newborn, residence, occupation, maternal education level, height, and family size), (2) maternal antepartum related factors (pregnancy status, parity, gestational age, nutritional status, ANC check-up, number of ANC visits, and previous history of adverse birth outcomes [Preterm birth, Low birth weight, still births], and medical illnesses during or prior to pregnancy), (3) maternal and fetal intrapartum related factors (fetal presentation at birth, mode of delivery, duration of labor, membrane status at birth, amniotic fluid color at birth, and birth weight).

### Data management and analysis

Data collected was edited, cleaned, entered into Epi-Data version 3.1 and exported into SPSS version 20 for statistical analysis. Correlation between independent variables was checked using variance inflation factor (VIF). Variables with P<0.25 in the bivariable logistic regression analysis were considered in the multivariable logistic regression model. Furthermore, model fitness was assessed using the Hosmer-Lemeshow goodness of fit test and omnibus tests of model coefficients before running the final model. Descriptive statistics were done and the results were presented with texts, tables, mean and standard deviation after the normality assumptions checked. A multivariable logistic regression analysis was done and the results were reported using adjusted odds ratios (OR) with 95% Confidence levels. Finally, a statistically significant level was declared at p <0.05 in the final regression model.

## Results

### Socio-demographic characteristics of participants

In this study, 345 participants were included, with a response rate of 96.6%. The mean age of the mothers was 26.92 years ± 4.7 SD with 123 (35.65%) of the mothers belonging to the age category of 20–24 years, and 52 (15%) of the mothers had short stature (<145cms). Besides, 222 (64.4%) of the mothers resided in rural areas. In addition, 193 (55.9%) of the mothers were housewives and only 92 (26.6%) of the women who participated in the study attended tertiary education (**Table 1**).

**Table 1:** Sociodemographic characteristics of the study participants, northeast Ethiopia, 2018.

List of variables	Frequency	Percentage %
**Age of the mother**		
15–19	11	3.1
20–24	123	35.65
25–29	110	31.88
30–34	84	24.34
≥35	17	4.92
**Marital status**		
Single	12	3.5
Married	333	96.5
**Residence of the mother**		
Urban	123	35.6
Rural	222	64.4
**Sex of newborn**		
Male	190	55
Female	155	45
**Height of the mother**		
<145 cm (short statured)	52	15.07
≥145 cm	293	84.92
**Occupation**		
house wife	193	55.9
Merchant	50	14.49
NGO employee	20	5.79
government employee	73	21.15
Others+	5	1.33
**Maternal Education level**		
No formal education	80	23.18
Primary	83	24.05
Secondary	90	26.08
Tertiary	92	26.66
**Family size (including extended family)**		
<5	233	67.5
≥5	112	32.5

Others+ = students, daily laborer, café servant

### Maternal and antepartum conditions of the mother

In this study, 311(90.14%) of the mothers carried singleton pregnancies and 206 (59.71%) of the mothers were primipara. In addition, 109 (31.58%) of the mothers had MUAC less than 22cm and 50 (14.5%) of the mothers gave birth with less than 37 weeks of gestation. Of the total participants, 34 (9.85%) of the mothers had experienced a medical illness during pregnancy and 335 (97.1%) of the mothers had ANC checkups during their respective pregnancy. Besides, only 135 (40.3%) of mothers had four and above ANC visits. However, only 24 (6.9%) of women had history of adverse birth outcomes prior to the current birth (**Table 2**).

**Table 2:** Maternal and antepartum related conditions of participants, northeast Ethiopia, 2018.

List of variables	Frequency	Percentage (%)
**Pregnancy status**		
Singleton	311	90.14
Multiple (≥2)	34	9.85
**Parity**		
Primi para	206	59.71
Multi para	139	40.28
**Gestational age (completed weeks)**		
<37	50	14.49
37–42	12	3.47
>42	283	82.02
**MUAC (in cm)**		
<22cm (malnourished)	109	31.58
≥22cm (well nourished)	236	68.4
**ANC check up**		
Yes	335	97.1
No	10	2.89
**Number of ANC visits (n = 335)**		
1–3 visits	200	59.7
4 and above visits	135	40.3
**Place where ANC follow-ups started**		
Government	220	63.7
Non-government	125	36.3
**previous adverse birth outcomes (i.e. abortion, PTB, LBW, Still birth)**		
Yes	24	6.9
No	321	93.1
**Any medical illness during pregnancy**		
Yes	34	9.85
No	311	90.14

### Intrapartum conditions of the mother

Results found that 224 (64.92%) of newborns were of cephalic presentation, 85 (24.63%) of labors were prolonged (>12 hours) and 50 (14.49%) of mothers had a problem of premature rupture of membranes. Finally, 73 (21.15%) mothers had complicated labor at birth and 95 (27.5%) of births were meconium stained at birth (**Table 3**).

**Table 3:** Prevalence and intrapartum conditions of the participants, northeast Ethiopia, 2018.

Characteristics	Frequency	Percentage (%)
**Presentation of the fetus at birth**		
Cephalic	224	64.92
None cephalic	121	35.07
**Mode of delivery**		
Spontaneous vaginal delivery	224	64.92
Caesarian section	121	35.07
**Induced labor**		
Yes	54	15.65
No	291	84.34
**Duration of Labor**		
>12 hours	85	24.63
≤12 hours	260	75.36
**Premature rupture of membrane**		
yes	50	14.49
no	295	85.50
**Complicated Labor**		
Yes	73	21.15
No	272	78.84
**Presence of meconium at birth**		
Yes	110	31.9
No	235	68.1
**Status of amniotic fluid at birth**		
Clear	235	68.1
Meconium Stained	81	23.5
Blood stained	29	8.4
**Birth asphyxia in the 1**^**st**^ **minute of birth (APGAR score <7)**		
Present	78	22.6
Absent	267	77.4
**Birth asphyxia in the 5**^**th**^ **minute of birth**		
Present	51	14.8
Absent	294	85.2
**Birth weight of newborn (in Kg)**		
<2.5	43	12.5
≥ 2.5	302	87.5

### Prevalence of birth asphyxia

The prevalence of birth asphyxia was found to be 22.6% [95% CI 19.2% - 26.4%] in the first minute and 14.8% (9.2–18%) in the fifth minute of birth based on APGAR scoring less than 7. Of these neonates with birth asphyxia, 70 (20.28%) were experienced with moderate birth asphyxia and 8 (2.32%) were experienced severe birth asphyxia (Table 3).

### Factors associated with birth asphyxia

The bi-variable logistic regression analysis showed that illness during pregnancy, primipara, gave birth through C-section, having blood-stained and meconium-stained amniotic fluid at birth, premature rupture of membrane, and presence of complication during labor were significantly associated with birth asphyxia. However, in the multivariable logistic regression model; being primipara, presenting with premature rupture of membranes, having complicated labor and/or presence of blood stained amniotic fluid at birth were the independent predictors for birth asphyxia. Besides, giving birth through C-section, and having meconium stained amniotic fluid at birth were not the independent predictors of birth asphyxia in the final model.

The likelihood of birth asphyxia among primipara mothers was 3.7 times greater compared to multipara mothers [AOR = 3.7: 95%CI 1.86–7.65]. Mothers with premature rupture membranes had 3.8 times greater risk of birth asphyxia compared to their counterparts [AOR = 3.85: 95% CI 1.76–8.45]. The odds of birth asphyxia among mothers which experienced complicated labor was more than threefold compared to those mothers who gave birth without any complications [AOR = 3.45: 95% CI 1.58–7.49]. Mothers with blood-stained amniotic fluid at birth were 5 times more likely to have a baby who experienced birth asphyxia compared to mothers who had clear amniotic fluid at birth [AOR = 5.02: 95% CI 1.69–14.87] (**Table 4**).

**Table 4:** Factors associated with birth asphyxia among mothers who gave birth at public hospitals, northeast Amhara, Ethiopia, 2018.

List of Variables	Birth Asphyxia		COR [95%CI]	AOR [95%CI]
	(yes)	(no)		
**ANC check up**				
Yes	72	263	1	1
No	6	4	5.47 (0.65, 46.06)	1.23 (0.53, 1.24)
**Illness during pregnancy**				
Yes	75	236	3.28(0.97–11.05)	2.74 (0.68–11.03)
No	3	31	1.00	1.00
**Parity**				
Primi Para	62	144	3.31(1.82–6.03)	3.77 (1.86–7.65)*
Multi Para	16	123	1.00	1.00
**Mode of delivery**				
SVD	41	183	1.00	1.00
C-Section	37	84	1.96 (1.17–3.287)	1.33(0.71–2.51)
**Premature rupture of membrane (PROM)**				
Yes	57	238	1.00	1.00
No	21	29	3.02 (1.60–5.68)	3.85 (1.76–8.45)**
**Complicated labor**				
Yes	35	38	4.90 (2.79–8.61)	3.45(1.58–7.49)**
No	43	229	1.00	1.00
**Status of amniotic fluid**				
Clear	27	208	1.00	1.00
Meconium stained	33	58	4.38(2.53–8.24)	2.35(0.97–5.68)
Blood stained	18	11	12.61 (5.44–30.25)	(1.69–14.87)***

* P<0.05

**P<0.01

***P<0.001

COR = Crude Odds Ratio, AOR = Adjusted Odds Ratio, CI = Confidence Interval, SVD = Spontaneous Vaginal Delivery, C-Section = Cesarean Section, Complicated labor: labor presented with either fever, prolonged labour, breech delivery, cord around child’s neck, premature delivery, or large baby size.

## Discussion

In developing countries, rates of birth asphyxia are several folds higher, ranging from 4.6 per 1000 to 26 per 1000 births and case fatality rates can be 40% or higher [29]. However, accurate epidemiological data is not adequate, and the exact burden of birth asphyxia in developing countries like Ethiopia is unknown. Studies conducted in Gondar University hospital, Jimma Zone, southwest Ethiopia and southern Ethiopia revealed that 12.5%, 47.5% and 26.2% respectively of neonatal mortality were attributed to birth asphyxia [30–32]. Moreover, the high prevalence of birth asphyxia in this study suggests that high neonatal mortality in Amhara region can be explained related to birth asphyxia. Therefore, birth asphyxia reduction needs attention in the second transformation plan (HSTP 2016–2020) [21] and the newborn survival strategy (2016–2020) in the region and as well as in the country [33] that both road map are expected to be done in the coming year

In this study, the prevalence of birth asphyxia was 22.6% in the 1^st^ minute of birth. This finding is lower compared to a study conducted in Debre Tabor Hospital, Ethiopia (29.9%) [34], Nigeria (30.1%) [11] and a study conducted in Zambia (23%) [9]. In addition, this finding is lower than a review study conducted in Federal Medical Centre, Birnin Kebbi, Nigeria (24.7%) [10]. The study conducted to assess prevalence of birth asphyxia among term neonates admitted at Korle-Bu Teaching Hospital (KBTH) in Accra (61.8%) [35] showed lower finding as well. This could be explained by differences in sociocultural characters among study participants involved in the studies. Furthermore, the lower prevalence of birth asphyxia in the current study could be justified by more than fifty percent of newborns’ mothers are housewives, which allow them to have for more self-care, such as visiting health facilities compared to women who work and have less time to engage in self-promoting behaviors. Moreover, housewives have a better exposure with community health workers and may have a better understanding in the area along with time they have to care their new born.

The prevalence of birth asphyxia was found to be higher compared to a five year review study conducted in DilChora referral hospital, Dire Dawa, Ethiopia, which examined causes of admission (12.5%) [13]. Similarly, the prevalence is higher compared to a study completed in Cameron (14.5%) [36]. These differences may be due to some of the mothers were referred from lower-level health facilities to get advanced care in hospitals because of their serious complications in labor. Besides, there is a shortage of skilled health care providers during delivery in government hospitals. This shortage of skilled health care providers especially skilled midwiferies in government hospitals can be explained by health professionals’ engagement in private clinics in order to generate additional income, beyond the salary they receive from the government. Therefore, the above-listed reasons may explain why the prevalence of birth asphyxia was found to be high in this study.

In this study, primipara mothers were found to have four times greater risk of birth asphyxia compared to multiparous women. This finding agrees with a study done in Rio Grande do Norte which identify being primipara is one of the risk factor at birth [18], and another study conducted in Pakistan which has the similar finding[16]. Similarly, a study conducted in central Tigray, Ethiopia indicated that primiparity is an independent risk factor of birth asphyxia [19]. Furthermore, a study conducted at public hospitals in Nigeria, revealed that primipara is one of the predictors of birth asphyxia [37]. Accordingly, primipara mothers tend to be found in the younger age bracket and they are more prone for mal-presentations and prolonged obstructed labor. Thus, it is believed that perinatal asphyxia is expected to be high among these women compared to the multipara women.

In addition, the majority (80%) of cases of perinatal asphyxia occur intrapartum or during labor [38]. Therefore, perinatal asphyxia can be caused by either maternal factors, uterine factors, cord factors and/or intrapartum infections (maternal fever in labor). In this study, mothers with premature rupture of membranes had 3.85 times greater risk of birth asphyxia compared to those who had a normal ruptured membrane. A prospective case-control study conducted on term neonates in a tertiary hospital in Yaoundé, Cameroon showed that there is significant association between premature ruptured membrane and birth asphyxia [36]. In rural areas, 14 percent of women had at least 4 antenatal care (ANC) visits compared to 46 percent in urban areas [7]. This could be a contributing factor for the late arrival of women into the health facilities and why some mothers experience premature rupture of membranes, which would put them at higher risk for birth asphyxia.

Additionally, the pathophysiology of birth asphyxia centers on the interruption of placental blood flow [3]. In this study, babies born with complicated labors were 3.45 times more likely to develop birth asphyxia compared to those born with uncomplicated labors. This finding is concurrent with a case control study conducted to identify socio-demographic and clinical risk factors associated with birth asphyxia in Matiari District of Sindh Province, Pakistan [17]. Moreover, this finding is consistent with a study conducted in Karachi Hospital, Pakistan [16], a study done in Dire Dawa referral hospital, Eastern Ethiopia [14], a study done in Nigeria Public Hospital, Nigeria [37], a prospective case-control study on term neonates in a tertiary hospital in Yaounde, Cameroon [36] and a study done in Jimma zone public hospitals, Southwest Ethiopia [25]. Births which result after a complicated labor, may be affected in several different ways, which may increase their risk and, result in vulnerable babies that are prone to asphyxia, as compared to uncomplicated labor. This can be due to the fact labor complications such as umbilical cord related problems, pre-eclampsia/eclampsia etc., may decrease the blood and oxygen supply to the infant and, in turn, lead to birth asphyxia.

Finally, in this study, the likelihood of birth asphyxia among babies born with blood stained amniotic fluid was found to be fivefold higher than compared to those born with clear amniotic fluid. An unmatched case control study conducted among live births at the Gondar, Ethiopia teaching hospital to identify the determinants of birth asphyxia confirmed that meconium stained amniotic fluid at birth is the independent predictor of birth asphyxia [39]. The study conducted in Debre Tabor Hospital, south Gondar, Ethiopia, which assessed neonatal asphyxia and associated factors among 154 births revealed that there was significant association between blood stained amniotic fluid and birth asphyxia [34]. A study conducted in KYAMC Hospital, which explored the determinants of birth asphyxia found there was significant relation between birth asphyxia and meconium stained amniotic fluid [15] and a case control study conducted in public hospitals of central Tigray, Ethiopia reported that meconium stained amniotic fluid was and independent risk factor of birth asphyxia [19]. Based on this, the presence of abnormal color of amniotic fluid indicates the risk of asphyxia and other infections. Subsequently, blood stained amniotic fluid appears to have high risk for birth asphyxia for newborns and babies may require intensive suctioning and resuscitation before they breathe independently.

### The authors acknowledged the following points as limitation of the study

The first limitation of this study may be that samples were collected at institution-based health care centers, which may not truly represent neonates delivered at home, health centers and other lower level health facilities. Thus, this may underestimate the prevalence depicted in the study. Secondly, the study did not use design effect. As a result, these conditions may affect the precision of the study and this may maximizing the standard error of the study. Finally, the study also shared the limitations of cross-sectional studies.

## Conclusions

In conclusion, the prevalence of birth asphyxia is comparable with previous studies conducted in developing countries, including Ethiopia. The multivariable logistic regression showed that being a primipara, having premature rupture of membranes, blood-stained amniotic fluid and/or labor complications were all independent predictors for birth asphyxia.

Therefore, health care providers, especially those working in labor and delivery wards, must give increased attention to complicated labors, to anticipate and take early action in order to avoid birth asphyxia. Additionally, mothers should be counseled regarding the health impact of premature rupture of membranes during antenatal care service delivery to reduce the burden of birth asphyxia. Lastly, government bodies should be urged to commit to the promotion and education about enhanced antenatal care services and their utilization among the community.

Further analytical studies supplemented by qualitative methods are recommended to establish causal relationship.

### Ethical issues and consent to participate

Ethical approval was obtained from the institutional ethical review board (IERB) of Wollo University, College of Health Sciences to ensure the potential benefit and scientific sound of the study. Permission letters were obtained from the administrative body of the hospitals. Finally, informed verbal consent was obtained from each participants including those mothers aged 15 years of age before interviewing them. In Ethiopian context, there was no need of assent for those women already married, as they have the right to decide on their own issues without parents’ interference. Thus, the ethical approval committee has taken into account the lower limit of marital age in the country. In addition, the respondents’ right to refuse or withdraw from participation was fully assured. The confidentiality and anonymity of the data was maintained by avoiding personal identifiers and using a locked personal computer.

## Supporting information

S1 File Questionnaire_Birth asphyxia_2018. (DOCX)

## Abbreviations

ANC: Antenatal Care
AOR: Adjusted Odds Ratio
APGAR: Appearance Pulse Grimace Activity Respiration
CI: Confidence Interval
COR: Crude Odds Ratio
HSTP: Health Sector Transformation Plan
IERB: Institutional Ethical Review Board
LBW: Low Birth Weight
MUAC: Mid-Upper Arm Circumference
NGO: Non-Governmental Organization
NICU: Neonatal Intensive Care Unit
PTB: Preterm Birth
SDG: Sustainable Development Goals
SVD: Spontaneous Vaginal Delivery
