# Supplementary material for: Birth asphyxia and its associated factors among newborns in public hospital, northeast Amhara, Ethiopia
Source: PLoS One. 2019 Dec 20;14(12):e0226891. doi: 10.1371/journal.pone.0226891 (PMC6924666; doi:10.1371/journal.pone.0226891)
Supplement: S1 File — (DOCX) [file pone.0226891.s001.docx]

## **ANNEX I: Consent Form for the Mothers**

1. **Information sheet**

Good morning? / Good afternoon? My name is ----------------------- I am a data collector and I am here on behalf of the researchers. I am going to ask you some questions about your history of pregnancy before you deliver the current child. The information we get from you helps as to recommend to the concerned body about the improvement of Antenatal, Delivery and Post-partum service. You were selected to participate in this study just by chance. The following are some general information about the study.

**The objective of the study:** To assess the magnitude of birth Asphyxia and associated factors among newborns in selected public Hospitals

**The benefit of the study**: there is no direct short term benefit for participants. However, it may use the policymakers to evaluate the service and help them to improve newborn and maternal health services.

**Risk of the study**: this study has no risk for participants.

**Right of the participants**: the participation is based on your voluntarism and you are not obligated to answer any question you do not wish to answer. This interview will take about 25-35 minutes. If you feel discomfort with the interview, please feel free to withdraw from the interview at any time.

**Confidentiality**: your name will not be written in this form and will never be used in connection with any information you tell us. All information given by you will be kept strictly confidential.

**Informed consent**

I have read this form or it has read to me in the language I understand all conditions stated above.

Therefore,

1. I agree to participate
2. I do not agree to participate

If the participant agrees to participate, skip to the next page.
If no, skip to the next participant by writing short reasons for refusal below.
………………………………………………………………………………………
Name of investigators: Abay W., Ayisheshum M., Christine St D.
Principal investigator: Abay Woday Tadesse

Address: Mob +251-9-11-37-68-50
E-mail: [abaywoday@yahoo.com](mailto:abaywoday@yahoo.com)
Supervisor address Tel …………………………….
Date of interview ………………………………….
Time started: ……………………………………….
Time completed …………………………………….
Name of data collector: …………………………………
Checked by:
Supervisor Name: ……………………… Sig……………………

Result of interview: ________ (fill either 1, 2, 3, or 4 as listed below)

Note: 1=Completed, 2=Respondent not available, 3=Refused to participate, 4=Partially completed

# **ANNEX-II English Version Questionnaire**

| Abay W, Ayisheshum M, Christine St D. prevalence and associated factors of birth asphyxia among newborns delivered at Public Hospitals in the Amhara Region, Ethiopia: A cross-sectional Study  Contact address of the Principal investigator: [abaywoday@yahoo.com](mailto:abaywoday@yahoo.com)  Cell phone: (+251) 0911376850 |
| --- |

**Identification related information**

Data collector’s Name: ______________________ Signature: ____________Date___________

Supervisor’s Name: ________________________ Signature: ___________________________

Questionnaire Code No: ____________________ Hospital Name: _______________________

**Part I: Now, I am going to ask some questions about your Background (socio-demographic and socioeconomic) information that is very important to the study.**

| ID code | Questions | Code of variables | Skip |
| --- | --- | --- | --- |
|  | How old is the mother in complete years? | _______ (in completed years) |  |
|  | What is the sex of the newborn? | 1. Male 2. Female |  |
|  | Where is your place of residence? | 1. Urban  2. Rural |  |
|  | What is your marital status? | 1. married /live together/ 2. divorced 3. widowed 4. single |  |
|  | Can you read and write simple sentences with any language you speak? | 1.Yes  2. No | Skip to Q107 |
|  | What was the highest level of education you have attended? | 1. Grade ………………… 2. Informal (X) ------------ |  |
|  | What is your main Occupation? | 1. Housewife 2. Farmer 3. merchant 4. Government employee 5. NGOs employee 6. Engaging small business/Micro 7. Others (Specify)___________ |  |
|  | What is your total Family size (including extended families)? | ____________(in number) |  |

**Part II: Now, I would like to ask some questions regarding your obstetrics and medical conditions in the last 9 months (Obstetric and Medical conditions).**

| Code | Questions | Code of variables | Skip |
| --- | --- | --- | --- |
|  | How many times you became pregnant including this birth? (abortion, alive birth, stillbirth, etc) | ________(put in number) |  |
|  | How many alive births do you have including the current birth? | ________(put in number) |  |
|  | Did you have ANC follow-ups for this pregnancy? | 1. Yes 2. No | Skip to Q208 |
|  | Where you start your ANC? | 1. Health post 2. Health center 3. Government Hospital 4. Private hospital 5. Private clinic 6. NGOs clinic 7. Other (specify)______________ |  |
|  | How many times you visit health facilities for ANC follow-ups only? | ________ (put in number) |  |
|  | Have you told as you have any danger symptoms of pregnancy during your ANC visits? | 1. Yes 2. No |  |
|  | If your answer is yes for Q207, Which danger symptoms do you have? (**multiple response possible** | 1. Severe headache 2. Blurred vision 3. Epigastric pain 4. Vaginal bleeding 5. Others (specify)__________ |  |
|  | Have you told as you had any medical problems in the last 9 months related to this pregnancy? | 1. Yes 2. No | Skip to Q210 |
|  | If your answer is yes for Q213, Which medical problems? (**multiple response possible** | 1. Diabetes mellitus 2. Hypertension 3. Cardiac problem 4. PROM 5. UTI 6. Others (specify)_________ |  |
|  | Do you have History of Adverse birth outcomes before this pregnancy? (Abortion, Low birth weight, Preterm birth, Small for gestational age, Still birth) | 1. Yes 2. No | Skip to Part III |
|  | If your answer is yes for Q210, which one of the followings? (**multiple response possible)** | 1. Preterm birth  2.. Low birth weight  3.. Abortion  4.. Still birth  5. SGA  6. Other (specify)………………… |  |

**Part III: the intrapartum conditions and newborn related questions**

| **ID Code** | **Questions** | **Code of variables** | **Skip** |
| --- | --- | --- | --- |
|  | What was the Presentation of the fetus at birth | 1. Cephalic 2. Non-cephalic |  |
|  | What of the mode of delivery this baby born? | 1. Spontaneous vaginal 2. C-section 3. Others (specify)__________ |  |
|  | What was the duration of the labor? | ---------------(put in hours) |  |
|  | What was the status of membrane at arrival? | 1. Intact 2. Ruptured |  |
|  | Is the presence of meconium seen during labor and delivery? | 1. Yes 2. No | Skip to Q310 |
|  | Status of amniotic fluid? | 1. Clear 2. Meconium stained 3. Bloody stained |  |
|  | Is there any complication during labor? | 1. Yes 2. No | Skip to Q310 |
|  | If Q305 is yes, which type of complication occurred? (**multiple response possible)** | 1. Pre-Eclampsia 2. Eclampsia 3. Excessive bleeding 4. Breech delivery 5. Cord around the fetal neck 6. Cord prolapse 7. Other (specify) _________ |  |

**Part IV: Questions to be filled from medical records or by Measurement**

1. What is the 1st minute APGAR score? ____________(put in number)
2. What is the 5th minute APGAR score? ____________(put in number)
3. What is the gestational age at birth? (**based on** **LMNP or see card for physician Diagnosis or see early ultrasound result)** ________ (put In completed weeks)
4. What is the Weight of the new born at birth (**measure or see cards**) __________ (put in kilo grams)
5. What is the height of the mother at birth (**measure**) ______ (**in centimeters**)
6. What is the MUAC of the mother at birth (**measure**) ______ (**in centimeters**)
7. What is the number of newborns at birth mother gave**? ______ (put in number)**

**Thank You!**
